# *Corynebacterium* Species Inhibit *Streptococcus pneumoniae* Colonization and Infection of the Mouse Airway

**DOI:** 10.3389/fmicb.2021.804935

**Published:** 2022-01-10

**Authors:** Kadi J. Horn, Alexander C. Jaberi Vivar, Vera Arenas, Sameer Andani, Edward N. Janoff, Sarah E. Clark

**Affiliations:** ^1^Department of Otolaryngology Head and Neck Surgery, University of Colorado School of Medicine, Aurora, CO, United States; ^2^Department of Developmental Biology, Washington University in St. Louis, St. Louis, MO, United States; ^3^Department of Molecular, Cell, and Developmental Biology, University of California, Los Angeles, Los Angeles, CA, United States; ^4^Division of Infectious Diseases, University of Colorado School of Medicine, Aurora, CO, United States; ^5^Denver Veterans Affairs Medical Center, Aurora, CO, United States

**Keywords:** *Corynebacterium*, *Streptococcus pneumoniae*, nasopharyngeal colonization, lung infection, pneumonia, pneumococcus, bacterial lipases

## Abstract

The stability and composition of the airway microbiome is an important determinant of respiratory health. Some airway bacteria are considered to be beneficial due to their potential to impede the acquisition and persistence of opportunistic bacterial pathogens such as *Streptococcus pneumoniae*. Among such organisms, the presence of *Corynebacterium* species correlates with reduced *S. pneumoniae* in both adults and children, in whom *Corynebacterium* abundance is predictive of *S. pneumoniae* infection risk. Previously, *Corynebacterium accolens* was shown to express a lipase which cleaves host lipids, resulting in the production of fatty acids that inhibit growth of *S. pneumoniae in vitro*. However, it was unclear whether this mechanism contributes to *Corynebacterium*-*S. pneumoniae* interactions *in vivo*. To address this question, we developed a mouse model for *Corynebacterium* colonization in which colonization with either *C. accolens* or another species, *Corynebacterium amycolatum*, significantly reduced *S. pneumoniae* acquisition in the upper airway and infection in the lung. Moreover, the lungs of co-infected mice had reduced pro-inflammatory cytokines and inflammatory myeloid cells, indicating resolution of infection-associated inflammation. The inhibitory effect of *C. accolens* on *S. pneumoniae in vivo* was mediated by lipase-dependent and independent effects, indicating that both this and other bacterial factors contribute to *Corynebacterium*-mediated protection in the airway. We also identified a previously uncharacterized bacterial lipase in *C. amycolatum* that is required for inhibition of *S. pneumoniae* growth *in vitro*. Together, these findings demonstrate the protective potential of airway *Corynebacterium* species and establish a new model for investigating the impact of commensal microbiota, such as *Corynebacterium*, on maintaining respiratory health.

## Introduction

Respiratory tract infections are a prominent a global health problem. Pneumonia, or lung infection, is a major source of mortality as the 4th most common cause of death worldwide (World Health Organization [WHO], 2021). Children are particularly vulnerable, as pneumonia is the leading cause of death in people under 5 years old (Centers for Disease Control and Prevention, 2019). In the United States, over one million emergency room visits are due to pneumonia every year (Centers for Disease Control and Prevention, 2019). The most common bacterial cause of pneumonia is the Gram-positive bacterial pathogen *Streptococcus pneumoniae*, also known as the pneumococcus (Jose et al., 2015; Torres et al., 2018). *S. pneumoniae* vaccines are extremely effective against invasive pneumococcal disease but are substantially less protective against pneumococcal pneumonia (Cutts et al., 2005; Pilishvili and Bennett, 2015), highlighting the need for new therapeutic strategies to reduce the burden of disease. *S. pneumoniae* is an opportunistic pathogen, as colonization in the nasopharynx of the upper airway is asymptomatic and susceptibility to infection differs among individuals. While this variation is not completely understood, the composition and function of the airway microbiome have emerged as important contributing factors (Clark, 2020). The disruption of the airway microbiome following antibiotic therapy correlates with an increased risk of *S. pneumoniae* acquisition (Biesbroek et al., 2014; Teo et al., 2015). Closely related *Streptococcus* commensals produce factors such as bacteriocins that kill *S. pneumoniae*, and serial administration of such commensals has shown some efficacy in reducing the incidence of pneumococcal-associated infections including otitis media in children (Marchisio et al., 2015; La Mantia et al., 2017). However, the contributions of other members of the commensal airway microbiome, particularly non-streptococcal species, to protection against *S. pneumoniae* are poorly understood.

Next-generation microbiome sequencing studies have identified additional members of the airway microbiome which are proposed to improve protection against pathogens including *S. pneumoniae*. Carriage of *Corynebacteriu*m, frequently together with *Dolosigranulum*, is associated with fewer upper and lower respiratory tract infections in both children and adults (Chonmaitree et al., 2017; Hasegawa et al., 2017; Copeland et al., 2018; Lappan et al., 2018; Man et al., 2019). *Corynebacterium* species are Gram-positive bacteria which frequently colonize the skin and upper airway. While *Corynebacterium diphtheriae*, the causative agent of diphtheria, is an important pathogen (Bernard, 2012), other *Corynebacterium* species are rarely associated with disease. Case reports have identified some *Corynebacterium* species associated with pneumonia, most frequently *Corynebacterium pseudodiphtheriticum* (Yang et al., 2018), indicating opportunistic infections may occur. However, non-*C. diphtheriae* species are more often associated with respiratory health, as the abundance of *Corynebacterium* species in nasopharynx correlates with reduced *S. pneumoniae* colonization and infection in several studies (Laufer et al., 2011; Pettigrew et al., 2012; Bomar et al., 2016; Kelly et al., 2017; Xu et al., 2021). Longitudinal studies tracking upper airway microbiome dynamics in children over time indicate that *Corynebacterium* abundance is predictive of both *S. pneumoniae* carriage and risk of respiratory tract infection (Biesbroek et al., 2014; Teo et al., 2015; Bosch et al., 2017; Kelly et al., 2021). Both children and adults with a higher abundance of *Corynebacterium* also have more stable airway microbiomes, compared to those with a predominance of opportunistic pathogens including *S. pneumoniae* (Bosch et al., 2017; Piters et al., 2019; Kelly et al., 2021). Together, these studies indicate that *Corynebacterium* species may play an important role in microbiome-mediated protection against pathogens such as *S. pneumoniae*, thereby reducing susceptibility to pneumonia.

Interruption of the potential protective benefit of *Corynebacterium* species in the airway is of particular concern for patients receiving antibiotics, as antibiotic therapy selectively reduces the *Corynebacterium* population while pathogens including *S. pneumoniae* are enriched (Teo et al., 2015; Prevaes et al., 2016; Kelly et al., 2021). Mechanistically, several *Corynebacterium* species can directly inhibit growth of *S. pneumoniae in vitro* (Bomar et al., 2016; Kelly et al., 2021; Xu et al., 2021). Among these, *Corynebacterium accolens* inhibits *S. pneumoniae* growth by expression of a bacterial lipase called LipS1, which hydrolyses host triacylglycerols, releasing free fatty acids that kill *S. pneumoniae* (Bomar et al., 2016). Fatty acids (FAs) are long-chain hydrocarbons with a terminal carboxyl group which are a major component of lipids. *Corynebacterium* species use fatty acids for growth, and lipophilic species including *C. accolens* require exogenous lipids to survive (Bernard, 2012; Tchoupa et al., 2021). Two non-lipophilic *Corynebacterium* species, which do not require host lipids for growth, were recently shown to inhibit *S. pneumoniae in vitro*, though the mechanisms were not identified (Xu et al., 2021). Another non-lipophilic species, *Corynebacterium amycolatum*, is frequently isolated from the human nasopharynx (Kaspar et al., 2016; Kelly et al., 2021) but is unusual in that it lacks mycolic acid, a long chain fatty acid which is a major component of the cell wall in almost all other *Corynebacterium* (Collins et al., 1988; Marrakchi et al., 2014). It is unclear whether FA production by bacterial lipases are universally required for *Corynebacterium*-mediated inhibition of *S. pneumoniae*, or whether diverse *Corynebacterium* species, including those less dependent on exogenous lipids, utilize similar mechanisms.

Understanding the host and bacterial requirements for *Corynebacterium*-mediated protection against *S. pneumoniae* is hampered by the lack of animal models for *Corynebacterium* colonization. Two related studies employing a five-dose serial inoculation of *C. pseudodiphtheriticum* in infant mice found that this strategy improves protection against *S. pneumoniae* (Moyano et al., 2020) as well as secondary pneumococcal infection in Respiratory Syncytial Virus (RSV)-infected mice (Kanmani et al., 2017). However, unknown is whether *C. pseudodiphtheriticum* actually colonizes the infant mouse airway in this model or if other *Corynebacterium* species can be used in a similar approach. For this study, we developed a mouse model to investigate the impact of two distinct species, *C. accolens* and *C. amycolatum*, on protection against *S. pneumoniae in vitro* and *in vivo*. Our findings indicate that both *C. accolens* and *C. amycolatum* can colonize the airways of antibiotic treated mice, and that pre-exposure to these *Corynebacterium* species protects against *S. pneumoniae* colonization of the upper airway as well as infection and inflammation in the lung. We envision that this co-infection model can be used to further understand how exposure to different *Corynebacterium* species in the airway influences pneumococcal colonization and disease.

## Materials and Methods

### Bacterial Strains

*Corynebacterium accolens* strain ATCC^®^ 49726™ was obtained from the American Type Culture Collection. *Corynebacterium amycolatum* strain SK46, HM-109 was obtained through BEI Resources, NIAID, NIH as part of the Human Microbiome Project. *Corynebacterium* strains were grown from glycerol stocks on BHI agar plates (BD Difco™ Bacto™ Brain Heart Infusion, Thermo Fisher Scientific) aerobically at 37°C overnight. Growth from fresh plates was used to inoculate BHI broth cultures, which were grown overnight aerobically at 37°C with shaking at 200 rpm in preparation for *in vitro* assays and *in vivo* infections. BHI plates and broth were supplemented with 1% Tween^®^ 80 (polysorbate, VWR) to provide an exogenous lipid source, unless otherwise specified. When indicated, 180 mg/mL triolein or oleic acid (VWR) were added to plates or broth cultures.

Streptomycin resistant variants of *Streptococcus pneumoniae* serotype 2 strain D39 and serotype 4 strain TIGR4 were kind gifts from Dr. Jeffrey N. Weiser (New York University). *S. pneumoniae* serotype 3 strain ATCC^®^ 6303™ from the American Type Culture Collection was also used. *S. pneumoniae* was grown in Todd Hewitt Broth supplemented with 5% Yeast Extract (BD Bacto™) and 50 μg/mL streptomycin (MilliporeSigma) for streptomycin^R^ strains. For all experiments, *S. pneumoniae* cultures were grown from frozen stocks at 37°C with 5% CO_2_ without shaking to mid-log phase, centrifuged at ≥ 20,000 × g for 10 min, and resuspended in phosphate-buffered saline (PBS). *S. pneumoniae* CFUs were determined by serial dilution plating on Tryptic Soy Broth agar (TSB, MP Biomedicals) supplemented with neomycin 5 μg/mL, streptomycin 50 μg/mL (streptomycin^R^ strains), and fresh catalase (5,000 Units/plate, Worthington Biomedical Corporation Lakewood, NJ) at 37°C with 5% CO_2_ overnight.

### Inhibition of *S. pneumoniae* Growth by Supernatant From *Corynebacterium* Species

For supernatant inhibition assays, *C. accolens* and *C. amycolatum* were grown on BHI agar supplemented with or without 1% Tween 80 and 180 mg/mL triolein. Triolein was emulsified in 100% ethanol for spreading onto plates, and plates were dried to evaporate ethanol prior to inoculation. Plates were incubated under aerobic conditions at 37°C overnight. Cultures were inoculated from fresh plates in BHI broth with or without 1% Tween 80 and 180 mg/mL triolein and grown aerobically at 37°C overnight with shaking at 200 rpm. OD_600_ was measured to standardize starting inocula, and cultures were centrifuged at ≥ 20,000 × g for 10 min to pellet bacterial cells. Cell-free supernatants (200 μL/plate) were spread onto TSB agar plates containing neomycin 5 μg/mL, streptomycin 50 μg/mL (streptomycin^R^ strains), and fresh catalase (5,000 Units/plate) and allowed to dry completely prior to *S. pneumoniae* serial dilution plating. For the dose response curve, supernatants were diluted in BHI broth (100–1.6% supernatant) prior to plating. *S. pneumoniae* serial dilutions were completed in PBS, with 10 μL spotted onto culture plates per dilution, plated in duplicate. Plates were incubated at 37°C with 5% CO_2_ overnight for *S. pneumoniae* CFU enumeration.

### Construction of *Corynebacterium* Mutant Strains

Mutation of the lipase encoding *lipS1* in *C. accolens* was performed as described previously (Bomar et al., 2016). Briefly, ∼1 kb regions flanking the coding sequence region for WP_005285206.1 in *C. accolens* ATCC 49726 (nucleotides 2,348,522–2,349,946) were amplified using Q5 high fidelity DNA polymerase (NEB) with primers designed to add a 21 bp scar sequence on the end of the 5′ fragment and beginning of the 3′ fragment, respectively, in addition to a stop codon at the end of the 3′ fragment (see Supplementary Table 1 for primer sequences). PCR products were purified using the Monarch PCR and DNA cleanup kit (NEB). We used the plasmid pKO (KAN) as a suicide vector for *lips1* cloning. pKO (KAN) was a gift from Markus Seeger (Addgene #110086; RRID:Addgene_110086).^1^ pKO was digested with *Pst*I and treated with calf intestinal phosphatase according to manufacturer’s instructions (NEB) and purified using the GeneJET plasmid miniprep kit (Thermo Fisher Scientific). Purified plasmid and PCR products were assembled by Gibson assembly using the HiFi DNA Assembly Cloning Kit (NEB), and the assembled sequence was transformed into high efficiency NEB 5-alpha competent *E. coli* (NEB). Clones containing the assembly sequence inserted into pKO were identified by sequencing (Quintara Biosciences, Hayward CA). Competent cells of *C. accolens* were prepared and pKO containing the *lipS1* flanking regions was introduced by electroporation as previously described for *Corynebacterium glutamicum* (Eggeling et al., 2005). Electroporated cells were resuspended in BHI Tween 80 broth containing sorbitol (9.1 g/100 mL) and heat shocked at 46°C for 6 min followed by incubation at 37°C for 1 h prior to plating on selective media. Transformants were grown on BHI agar supplemented with Tween 80, sorbitol, and kanamycin 20 μg/mL to select for clones which integrated the *lipS1* flanking regions and kanamycin resistance gene from the modified pKO plasmid into the *C. accolens* genome by homologous recombination (kanamycin^R^) followed by 5% sucrose to select for clones which lost the plasmid vector backbone (sucrose selection against *sacB* in pKO). Deletions in *lipS1* were confirmed by PCR amplification and sequencing (Quintara Biosciences, Hayward CA). The same process was used to construct an in-frame deletion in the coding sequence region for WP_005510233.1 in *C. amycolatum* (nucleotides 16,770–17,957) using primers specific for the flanking sequence regions (Supplementary Table 1).

### Animals

Male and female 6–12 week old C57BL/6J (WT) mice, purchased from The Jackson Laboratory (RRID:IMSR_JAX:000664), were bred in-house. Mouse colonies were maintained in the University of Colorado Office of Laboratory Animal Resources.

### Infections

All mice were treated with ingested broad-spectrum antibiotics (ampicillin 1 g/L, neomycin 1 g/L, metronidazole 1 g/L, vancomycin 0.5 g/L, MilliporeSigma and McKesson) *ad libitum* for 7 days until 48 h prior to infection. Bacterial loads in the stool pre- vs. post-antibiotic treatment were detected by qPCR as described previously (Ricchi et al., 2017). Briefly, stools were weighed and genomic DNA was extracted using the PureLink™ Genomic DNA Mini Kit (Thermo Fisher Scientific). Quantitative PCR was performed using iTaq™ Universal SYBR^®^ Green Supermix (BioRad) together with 200 nM forward and reverse primers (Supplementary Table 1; Jimeno et al., 2018) and 2.5 μL template DNA. Reactions were performed using a CFX Connect™ Real-Time System (BioRad) with cycling conditions of: (1) 94°C for 4 min; (2) 40 cycles of 15 s at 95°C, 30 s at 60°C, and (3) 72°C for 10 min. A standard curve was generated using a known concentration of *S. pneumoniae* D39 gDNA, which together with the input ng/μL DNA and Ct values was used to calculate total 16S rRNA gene copy number as described previously (Gomes-Neto et al., 2017). qPCR data was analyzed using CFX Manager Software (version 2.1, BioRad). Nasal lavages, which can only be collected upon sacrifice, were obtained from separate groups of mice with and without antibiotic treatment for quantification of bacterial load by qPCR as above. Lavages were performed by the instillation of 200 μL PBS into cannulated trachea through the nasal cavity and collected from the nares.

Mice were infected with *C. accolens*, *C. amycolatum*, and/or *S. pneumoniae*, as indicated, at 10^7^ CFU/mouse i.n. under inhaled isoflurane anesthesia. Nasal lavages were collected as above. Lungs were homogenized using a Bullet Blender tissue homogenizer (Stellar Scientific, Baltimore, MD). *S. pneumoniae* nasal lavage and lung CFUs were calculated following serial dilution and growth on Tryptic Soy agar plates containing neomycin (5 μg/mL, MilliporeSigma) and streptomycin (50 μg/mL, only streptomycin^R^ strains), supplemented with fresh catalase (5,000 Units/plate, Worthington Biochemical Corporation, Lakewood, NJ) grown overnight at 37°C with 5% CO_2_. *Corynebacterium* CFUs were calculated following serial dilution and growth on BHI plates containing 1% Tween 80 grown overnight aerobically at 37°C. In co-infected mice, *Corynebacterium* CFUs were determined by subtracting *S. pneumoniae* CFUs (on streptomycin plates) from total growth (on non-selective BHI plates).

### Flow Cytometry

Single cells were prepared from lungs following transcardial perfusion with PBS as described previously (Clark et al., 2020). Briefly, lungs were minced and digested with DNAseI (30 μg/mL, MilliporeSigma) and type 4 collagenase (1 mg/mL, Worthington Biochemical Corporation). Lung preparations were filtered with a 70 μM strainer prior to lysis of red blood cells by brief (1 min) incubation in RBC lysis buffer (0.15M NH_4_Cl, 10 mM KHCO3, 0.1 mM Na_2_EDTA, pH 7.4). Cell Fc receptors were blocked with anti-CD16/32 2.4G2 hybridoma supernatant prior to staining in FACS buffer (1% BSA, 0.01% NaN_3_ in PBS). Cells were fixed in 1% paraformaldehyde. Antibodies used included: Siglec F (clone E50-2440), MHCII (clone M5/114.15.2), Ly6G (clone 1A8), Ly6C (clone HK1.4), CD45.2 (clone 104), CD11c (clone N418), CD11b (clone M1/70), CD64 (clone X54-5/7.1), and CD103 (clone 2E7). Antibodies were purchased from eBioscience, BD, BioLegend, or Thermo Fisher Scientific. Flow cytometry was performed using an LSRII (BD) in the Rocky Mountain Regional VAMC Research Core, Aurora CO and data were analyzed using FlowJo™ v10.8 Software (BD Life Sciences).

### Cytokine and Chemokine Analysis

BAL cytokines and chemokines were measured using a LEGENDplex™ Mouse Inflammation Panel, and data were analyzed using the LEGENDplex™ Data Analysis Software Suite (BioLegend). Analytes were detected on the LSR Fortessa X-20 in the ImmunoMicro Flow Cytometry Shared Resource Laboratory at the University of Colorado Anschutz Medical Campus (RRID:SCR_021321). Serum cytokines were measured using mouse TNFα and IFNγ ELISA kits (BD), with analytes detected on a Synergy™ HT Microplate Reader (BioTek).

### Mass Spectrometry for Detection of Fatty Acids

Samples were analyzed via ultra-high pressure liquid chromatography coupled to mass spectrometry (UHPLC-MS) at the University of Colorado School of Medicine Metabolomics Core. Nasal lavages were vigorously vortexed to homogenize, then diluted 1:1 in Optima LC-MS (Fisher) grade ice-cold MeOH and vortexed briefly again. Next, samples were incubated at –20°C for 30 min, then centrifuged at 4°C, 30,130 × g for 10 min to remove remaining solids as previously described (Nemkov et al., 2017; Reisz et al., 2019). Supernatants were transferred to pre-cooled autosampler vials, randomized, and analyzed on a Vanquish UHPLC coupled to a Q Exactive MS (Thermo Fisher Scientific, San Jose, CA, United States). Extracts (15 μL) were resolved with flow rate of 300–400 μL/min over an Acuity HSS T3 column (150 × 2.1 mm, 1.8 μm, Waters, Milford, MA, United States) with a 17-min gradient in negative ion polarity mode as previously described (Reisz et al., 2019). Samples were introduced to the MS via electrospray ionization with the MS scanning in full MS mode over the range of 150–1,500 m/z. As a quality control measure, technical replicates were injected every six samples to verify instrument stability (Nemkov et al., 2019). Fatty acids were annotated and integrated with Maven (Princeton University) in reference to the KEGG database. Peak quality was validated using blanks, technical mixes, and ^13^C natural isotope abundance (Nemkov et al., 2015).

### Study Approval

These studies were approved by the Animal Care and Use Committee (Protocol #00927) and the Institutional Biosafety Committee (Protocol #1418) of the University of Colorado School of Medicine.

### Statistical Analysis

Graphing and statistical analysis were completed using GraphPad Prism (Version 8.4.3, GraphPad Software, LLC, San Diego, CA). Statistical tests included *t*-tests for single comparisons, ANOVA with multiple group comparisons of normally distributed data and Kruskal-Wallis tests with multiple group comparisons of non-normal (non-Gaussian) distributions of infectious burden data (due to the limit of detection cut-off). For all statistical tests *p* < 0.05 was considered significant. All *in vivo* infections were conducted with 3–6 mice per group and repeated 3 times. For *in vitro* assays, experiments were completed with 2–3 technical replicates per condition and repeated 3 times.

## Results

### Inhibition of *S. pneumoniae* Growth *in vitro* by Lipophilic and Non-lipophilic *Corynebacterium* Species

**FIGURE 1:**
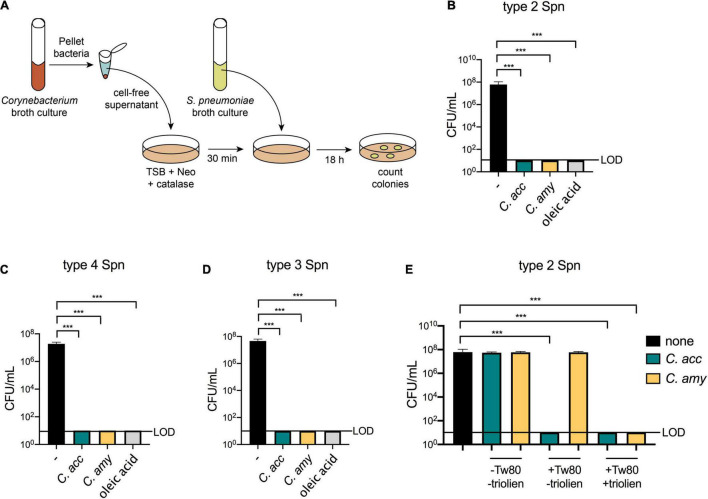
Inhibition of *S. pneumoniae* growth *in vitro* by lipophilic and non-lipophilic *Corynebacterium* species. Supernatant inhibition assay schematic using tryptic soy broth (TSB), neomycin (Neo), and catalase plates **(A)**. Growth of type 2 *S. pneumoniae* (Spn), **(B)**, type 4 Spn **(C)**, and type 3 Spn **(D)** on untreated plates (–) or on plates pre-treated with supernatants from *C. accolens* (*C. acc*) or *C. amycolatum* (*C. amy*) or with 180 mg/mL oleic acid. Growth of type 2 Spn on untreated plates (–) or on plates pre-treated with supernatants from *C. acc* or *C. amy* grown in BHI with or without 1% Tween 80 and 180 mg/mL triolein **(E)**. Limit of detection (LOD) is indicated. Data are pooled from three independent experiments in duplicate. ***p < 0.001, ANOVA.

A recent survey of morphologically distinct *Corynebacterium* strains collected from infants showed that cell-free supernatants from four different lipophilic species, including *C. accolens*, inhibited *S. pneumoniae* growth *in vitro* (Kelly et al., 2021). However, supernatants from only 20% of the *Corynebacterium* isolates tested in this study were inhibitory, indicating that not all *Corynebacterium* strains mediate *S. pneumoniae* growth restriction *in vitro*. We considered whether *C. amycolatum*, which unlike *C. accolens* does not require exogenous FAs for either growth or mycolic acid production, inhibits *S. pneumoniae* growth *in vitro*. Cell-free supernatants from *C. accolens* or *C. amycolatum* were spread onto culture plates prior to *S. pneumoniae* inoculation, and *S. pneumoniae* colony forming units (CFUs) were determined following overnight incubation (Figure 1A). In contrast to robust growth of type 2 *S. pneumoniae* (strain D39) on untreated culture plates, no growth of *S. pneumoniae* was detected on plates pre-treated with supernatants from either *C. accolens* or *C. amycolatum* (Figure 1B and Supplementary Figure 1A). *S. pneumoniae* growth was also not detected on plates treated with oleic acid, a FA that kills *S. pneumoniae* (Clementi et al., 2013), included as a positive control. Supernatants from both *C. accolens* and *C. amycolatum* demonstrated similar inhibition of type 4 and type 3 *S. pneumoniae* strains (Figures 1C,D), indicating that multiple serotypes of *S. pneumoniae* are vulnerable to *C. accolens* and *C. amycolatum*-mediated growth restriction. Thus, both *C. accolens* and the non-lipophilic *C. amycolatum* inhibit *S. pneumoniae* growth *in vitro*.

We next considered whether the source or amount of FAs determined *Corynebacterium-*mediated inhibition of *S. pneumoniae* growth. In the supernatant assays described above, *Corynebacterium* cultures were supplemented with the synthetic surfactant Tween 80 and triolein, a naturally occurring triacylglycerol. Both Tween 80 and triolein are hydrolyzed by bacterial enzymes, resulting in the release of oleic acid, which is used by *Corynebacterium* as a carbon source for growth (Plou et al., 1998; Taoka et al., 2011). Triolein is hydrolyzed by bacterial lipases, while Tween 80 can be hydrolyzed by both lipases and esterases (Plou et al., 1998). As a result, the bacterial lipase LipS1 is required for growth of lipophilic *C. accolens* in triolein as the sole source of FAs, whereas other enzymes suffice for growth in Tween 80 (Bomar et al., 2016). To compare the relative importance of Tween 80 vs. triolein for *Corynebacterium*-mediated inhibition of *S. pneumoniae* growth, we investigated the inhibitory capacity of supernatants from *Corynebacterium* grown in BHI supplemented with none, one, or both of these lipid sources. For *C. accolens*, the addition of Tween 80 to cultures was sufficient for supernatant inhibition of *S. pneumoniae*. In contrast, *C. amycolatum* inhibition required the addition of both Tween 80 and triolein (Figure 1E). Titration of the amount of *Corynebacterium* supernatants added to culture plates revealed that at lower concentrations, *C. accolens* supernatants from cultures with triolein were more inhibitory than those without triolein (Supplementary Figure 1B). For *C. amycolatum*, triolein was required for inhibition, which only occurred when supernatants were added above a threshold concentration (Supplementary Figure 1B). We also confirmed that BHI alone, supplemented with Tween 80 or Tween 80 and triolein, was not inhibitory (Supplementary Figure 1B). These results indicate a dose-dependence for *C. accolens* and *C. amycolatum*-mediated inhibition of *S. pneumoniae*. In both cases, the inclusion of triolein increased the inhibitory effect, although the efficiency of *C. amycolatum*-mediated inhibition was lower than that of *C. accolens*, which was inhibitory in the presence of Tween 80 alone. This difference in substrate usage likely relates to the composition and expression of bacterial lipases and esterases responsible for triolein vs. Tween 80 cleavage in *C. amycolatum* compared with *C. accolens*. Therefore, despite the ability of *C. amycolatum* to grow without an external source of FAs, these are required for the inhibitory effect on *S. pneumoniae*. In aggregate, these results confirm and extend the importance of FA release following lipid hydrolysis for *Corynebacterium*-mediated inhibition of *S. pneumoniae in vitro* and identify differential requirements in distinct *Corynebacterium* species.

### *Corynebacterium* Species Colonize the Upper and Lower Airway of Antibiotic Treated Mice

**FIGURE 2:**
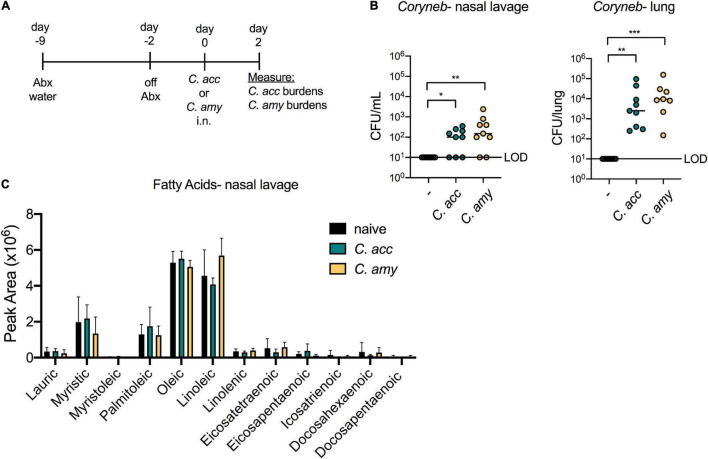
*Corynebacterium* species colonize the upper and lower airway of antibiotic treated mice. Timeline for antibiotic treatment followed by infection with *C. acc* or *C. amy* **(A)**. Burden of *Corynebacterium* (*Coryneb*) in the nasal lavage fluid and lungs of mice 48 h post-infection with *C. acc*, *C. amy*, or PBS (–) detected by live growth **(B)**. Relative abundance of fatty acids detected in the nasal lavage fluid of naïve mice and mice 48 h post-infection with *C. acc* or *C. amy* **(C)**. Data are pooled from three independent experiments with 3–6 mice per group. No significant differences detected in relative FA abundances between groups, ANOVA. *p < 0.05, **p < 0.01, ***p < 0.001, Kruskal-Wallis test.

In order to investigate whether *Corynebacterium* species mediate inhibition of *S. pneumoniae in vivo*, we first established a model of short-term *Corynebacterium* colonization. Mice were treated for 1 week with water containing an antibiotic cocktail to reduce the endogenous flora. One week of antibiotic treatment significantly decreased the total microbial burden in both the upper airway and gut (Supplementary Figure 2). Mice were taken off antibiotics 2 days prior to intranasal (i.n.) infection with *C. accolens*, *C. amycolatum*, or PBS (control), and *Corynebacterium* burdens were determined 2 days following infection (Figure 2A). Both *C. accolens* and *C. amycolatum* were detected in the upper airway (nasal lavage) and lungs of mice 48 h post-infection as measured by live CFU counts from nasal lavage fluid and homogenized lung tissue of mice infected with *Corynebacterium* species, but not PBS-treated controls (Figure 2B). Both sites were successfully colonized, albeit with generally higher numbers in the lungs than in the upper airway. These data indicate that both lipophilic and non-lipophilic *Corynebacterium* species can successfully colonize the airway of antibiotic-treated mice. In order to determine the availability of FAs in the murine upper airway, as well as whether *Corynebacterium* colonization influenced FA content, nasal lavage fluids were analyzed by mass spectrometry (MS). Several FAs were detected in the nasal lavage fluid, including oleic acid, myristic acid, and palmitoleic acid (Figure 2C), all of which are also present in human nasal lavage fluid (Do et al., 2008). These data are consistent with the presence of FAs in the upper airway of mice which are capable of supporting *Corynebacterium* growth. However, *Corynebacterium* colonization did not significantly alter the relative abundances of any of the FAs detected (Figure 2C). These data suggest that either short-term colonization is not sufficient to alter the FA landscape, or that FA turnover by host or bacterial metabolic processes obscured any temporal changes. This model for short-term *Corynebacterium* colonization of the murine airway provided the opportunity to characterize the physiologic and pathologic impact of these commensals on pneumococcal infection.

### Inflammatory Myeloid Cells Are Recruited to the Lungs of C*orynebacterium*-Colonized Mice

**FIGURE 3:**
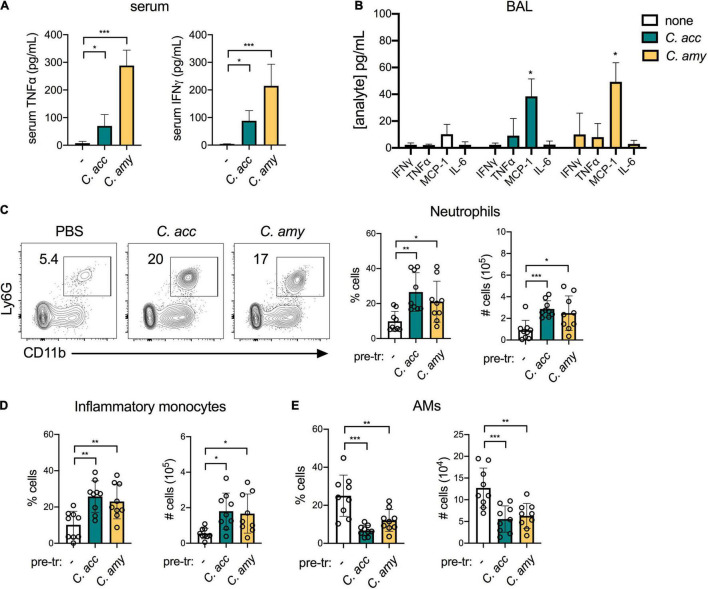
Inflammatory myeloid cells are recruited to the lungs of C*orynebacterium*-colonized mice. Systemic TNFα and IFNγ detected in mice 48 h post-infection with *C. acc*, *C. amy*, or PBS (–) **(A)**. Cytokines and chemokines detected in lung BAL in mice 48 h post-infection with *C. acc* or *C. amy* **(B)**, with significance indicated relative to mice pre-exposed to PBS (none). Percentage and total number of neutrophils (CD45^+^SiglecF^–^Ly6G^+^CD11b^+^ cells) **(C)**, inflammatory monocytes (CD45^+^ Ly6G^–^Ly6C^+^CD11b^+^ cells) **(D)**, and alveolar macrophages (AMs, CD45^+^SiglecF^+^CD11b^low^CD11c^+^ cells) **(E)** detected by flow cytometry in the lungs of mice 48 h post-infection with *C. acc*, *C. amy*, or PBS (–). Data are pooled from three independent experiments with 3–6 mice per group. *p < 0.05, **p < 0.01, ***p < 0.001, ANOVA.

We next interrogated the impact of short-term *Corynebacterium* colonization on local and systemic cytokine production as well as the recruitment of inflammatory myeloid cells to the lung. Both *C. accolens* and *C. amycolatum* induced a significant increase in systemic levels of the pro-inflammatory cytokines TNFα and IFNγ, compared with PBS-treated mice (Figure 3A). In the lung, however, we found that *Corynebacterium* colonization had little impact on cytokine expression in the bronchoalveolar lavage fluid (BAL) at 48 h post-infection, as measured by a 13-plex inflammatory cytokine/chemokine array. Of the 13 cytokines and chemokines tested, only MCP-1 (CCL2), an important chemokine for monocyte and macrophage recruitment (Gschwandtner et al., 2019), was significantly upregulated in the BAL of *C. accolens* and *C. amycolatum*-colonized mice (Figure 3B and not shown). These findings indicate that *Corynebacterium* colonization is associated with increased circulating pro-inflammatory cytokines, but a minimal such response in the lung.

We determined the impact of *Corynebacterium* colonization on lung innate immune cell populations by flow cytometry (see gating strategy, Supplementary Figure 3). *Corynebacterium* colonization was associated with an increase of both neutrophils (CD45^+^Ly6G^+^CD11b^+^ cells) and inflammatory monocytes (CD45^+^Ly6G^–^Ly6C^+^CD11b^+^ cells) in the lung (Figures 3C,D). These increases parallel the elevated MCP-1, which mobilizes the recruitment of inflammatory monocytes from the bone marrow (Gschwandtner et al., 2019), detected in the BAL of *Corynebacterium*-infected mice. Neutrophils are also recruited to the lung from the bone marrow, though responses to other chemokines such as MIP-1 (CXCL2) (Lin and Fessler, 2021) were not measured here. In contrast, the lungs of mice infected with *C. accolens* or *C. amycolatum* had reduced alveolar macrophages (AMs, CD45^+^SiglecF^+^CD11b^low^CD11c^+^ cells), compared with PBS treated controls (Figure 3E). Unlike neutrophils and inflammatory monocytes, AMs are largely self-renewing locally in the lung. As the first immune cell to encounter inhaled particles, AMs frequently undergo cell death following bacterial uptake (FitzGerald et al., 2020), consistent with our observation of reduced AMs in the lungs of *Corynebacterium* infected mice. We detected no changes in CD103^+^ dendritic cells (DCs, CD45^+^MHCII^+^SiglecF^–^CD11c^+^CD64^–^ cells) or CD11b^hi^ DCs, or the expression of MHCII in either population as an indicator of activation, in *Corynebacterium*-infected mice compared with PBS-treated controls (Supplementary Figure 4). Collectively, these data indicate that *Corynebacterium* colonization is associated with selective mucosal responses, including increased chemokine-associated myeloid cell recruitment to the lung but minimal changes to the local pro-inflammatory cytokine landscape.

### Colonization With *Corynebacterium* Inhibits Colonization and Infection With *S. pneumoniae in vivo*

**FIGURE 4:**
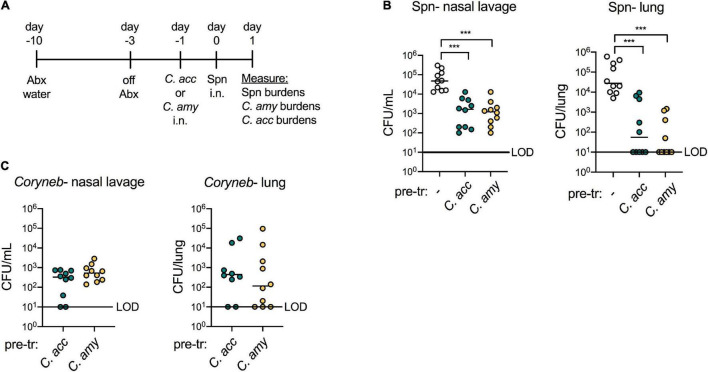
Colonization with *Corynebacterium* inhibits colonization and infection with *S. pneumoniae in vivo.* Timeline for antibiotic treatment followed by pre-exposure to PBS (–), *C. acc* or *C. amy* prior to infection with *S. pneumoniae* (Spn) **(A)**. Burden of Spn **(B)** or *Corynebacterium* (*Coryneb*) **(C)** in nasal lavage fluid and lungs of mice pre-exposed to PBS (–), *C. acc* or *C. amy* at 24 h post-Spn infection detected by live growth. Limit of detection (LOD) is indicated. Data are pooled from three independent experiments with 3–5 mice per group. ***p < 0.001, Kruskal-Wallis test.

Our goal in establishing a model for *Corynebacterium* colonization was to evaluate whether *Corynebacterium* species inhibit the acquisition and growth of *S. pneumoniae in vivo*, similar to *Corynebacterium*-mediated inhibition of *S. pneumoniae* observed by our group and others *in vitro*. We exposed antibiotic-treated mice to *C. accolens* or *C. amycolatum* 1 day prior to intranasal infection with streptomycin-resistant type 2 *S. pneumoniae* and plated nasal lavage contents and lung homogenates on selective media containing streptomycin 1 day later (Figure 4A). Compared with mice pre-exposed to PBS, colonization with either *C. accolens* or *C. amycolatum* significantly reduced the burden of *S. pneumoniae* in both the upper airway (nasal lavage) and lung by 24 h post-infection (Figure 4B). In mice pre-exposed to *Corynebacterium*, *S. pneumoniae* burdens were reduced by ∼1 log in the upper airway, and *S. pneumoniae* was not detected in the lungs of 50–60% of the animals in each *Corynebacterium* exposure group. *C. accolens* and *C. amycolatum* burdens remained detectable following growth in non-selective media in the majority of co-infected mice at this timepoint (Figure 4C). Further, mice with the highest *Corynebacterium* burdens had the lowest, frequently undetectable, *S. pneumoniae* burdens in the lung (Supplementary Figures 5A,B). Our data are consistent with longitudinal microbiome sequencing studies which suggest that *Corynebacterium* abundance predicts *S. pneumoniae* acquisition and infection risk in children (Biesbroek et al., 2014; Teo et al., 2015; Bosch et al., 2017; Kelly et al., 2021). Of particular note, these studies demonstrate the potential for live animal modeling of *Corynebacterium-S. pneumoniae* dynamics *in vivo* in order to elucidate the bacterial and host requirements for *Corynebacterium-*mediated protection against respiratory tract infection.

### Reduced Inflammation and Inflammatory Myeloid Cell Recruitment in the Lungs of *Corynebacterium-S. pneumoniae* Co-infected Mice

**FIGURE 5:**
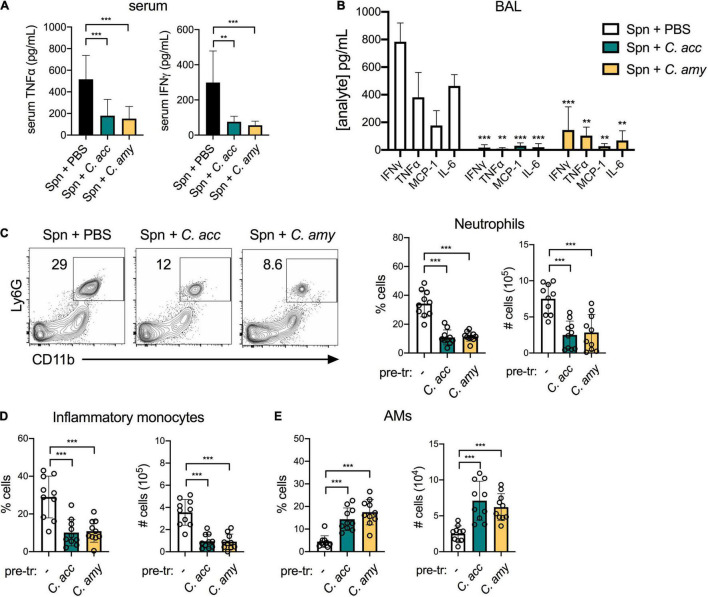
Reduced inflammation and inflammatory myeloid cell recruitment in the lungs of *Corynebacterium-S. pneumoniae* co-infected mice. Systemic TNFα and IFNγ detected in mice pre-exposed to PBS (–), *C. acc* or *C. amy* at 24 h post-*S. pneumoniae* (Spn) infection **(A)**. Cytokines and chemokines detected in lung BAL in mice pre-exposed to PBS (–), *C. acc* or *C. amy* at 24 h post-Spn infection **(B)**, significance indicated relative to mice pre-exposed to PBS (none). Percentage and total number of neutrophils **(C)**, inflammatory monocytes **(D)**, and alveolar macrophages (AMs) **(E)** detected by flow cytometry in the lungs of mice pre-exposed to PBS (–), *C. acc* or *C. amy* at 24 h post-Spn infection. Data are pooled from three independent experiments with 3–5 mice per group. **p < 0.01, ***p < 0.001, ANOVA.

*Corynebacterium* co-infected mice also had a reduced inflammatory response in the lung compared to mice infected with *S. pneumoniae* alone. In contrast to *S. pneumoniae* challenged mice pre-exposed to PBS, systemic TNFα and IFNγ were reduced in mice pre-exposed to *C. accolens* or *C. amycolatum* (Figure 5A). Similarly, in the BAL of co-infected mice, levels of several pro-inflammatory cytokines including TNFα, IFNγ, and IL-6 as well as the chemokine MCP-1 were reduced in mice pre-exposed to *Corynebacterium* compared to mice infected with *S. pneumoniae* alone (Figure 5B), while 9 other cytokines were not affected (not shown). Total neutrophils and inflammatory monocytes in the lungs of co-infected mice were also reduced compared with mice infected with *S. pneumoniae* alone (Figures 5C,D). In contrast, AMs were increased in co-infected mice (Figure 5E). Overall populations of lung DCs, which are important for mobilizing the adaptive immune response but may also facilitate pneumococcal dissemination (Rosendahl et al., 2013), remained the same, although CD103^+^ DC expression of the activation marker MHCII was significantly reduced in *Corynebacterium* co-infected mice compared to mice infected with *S. pneumoniae* alone (Supplementary Figure 6). The reduction in inflammatory myeloid cells together with the recovery of AMs, which are important for the resolution of inflammation in the lung, is consistent with a pro-resolving immune response in the lungs of co-infected mice. We conclude that colonization with either *C. accolens* or *C. amycolatum* improves early clearance of *S. pneumoniae* from the upper and lower airways of mice, in association with reduced, rather than enhanced, inflammation in the lung, likely limiting lung injury.

### *Corynebacterium* Lipases Contribute to, but Are Not Required for, Protection Against *S. pneumoniae in vivo*

We next sought to confirm the importance of the lipase LipS1 for *C. accolens*-mediated inhibition of *S. pneumoniae* growth *in vitro* and to determine whether *C. amycolatum* expresses a lipase with similar activity. We first constructed a *lipS1* mutant in our strain of *C. accolens* by in-frame gene deletion. As described for the original Δ*lipS1 C. accolens*, our Δ*lipS1 C. accolens* grew poorly in triolein as the sole source of FAs (Bomar et al., 2016), indicating LipS1 as critical for lipase-mediated hydrolysis and growth in *C. accolens* (Supplementary Figure 7A). In contrast, growth of Δ*lipS1 C. accolens* in Tween 80 was not impaired relative to wild-type (WT) *C. accolens*, presumably due to the hydrolysis of Tween 80 by bacterial esterases.

**FIGURE 6:**
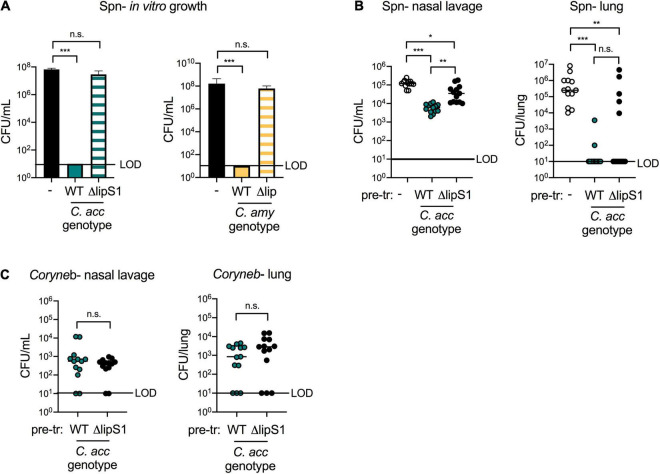
*Corynebacterium* lipases contribute to, but are not required for, protection against *S. pneumoniae in vivo.* Growth of type 2 *S. pneumoniae* (Spn) on untreated plates (–) or on plates pre-treated with supernatants from WT vs. *ΔlipS1 C. acc* or WT vs. *Δlip C. amy* grown in BHI with 1% Tween 80 and 180 mg/mL triolein **(A)**. Burden of Spn **(B)** or *Corynebacterium* (*Coryneb*) **(C)** in nasal lavage fluid and lungs of mice pre-exposed to PBS (–), WT *C. acc* or *ΔlipS1 C. acc* at 24 h post-Spn infection detected by live growth. Limit of detection (LOD) is indicated. Data are pooled from three independent experiments with 3–5 mice per group. *p < 0.05, **p < 0.01, ***p < 0.001, ANOVA **(A)** or Kruskal-Wallis test **(B,C)**.

Bacterial lipases in both *C. accolens* and *C. amycolatum* are essential for *Corynebacterium*-mediated inhibition of *S. pneumoniae* growth *in vitro* but have distinct characteristics. Supernatants from Δ*lipS1 C. accolens* did not inhibit *S. pneumoniae* growth *in vitro*, whereas those from WT *C. accolens* did (Figure 6A), confirming the importance of LipS1 for *S. pneumoniae* inhibition *in vitro*. A search for similar bacterial lipases in *C. amycolatum* in the NCBI database identified the protein WP_005510233.1 as sharing the greatest homology with that of *C. accolens* LipS1 (WP_005285206.1). Though overall protein sequence homology to *C. accolens* LipS1 was low (36%), the NCBI Conserved Domain Database (CDD, version cdd.v.3.19) (Lu et al., 2019) classified WP_005510233.1 as a member of the LIP family of secretory lipases with an *E*-value of 2.94e^–33^, similar to *C. accolens* LipS1. *C. amycolatum* WP_005510233.1 also contains the lipase motif G-X-S-X-G with a predicted active site serine residue (Jaeger et al., 1994), a shared feature among bacterial lipases. Analysis using the SignalP program (version 5.0) (Petersen et al., 2011) indicated that while both *C. accolens* LipS1 and *C. amycolatum* WP_005510233.1 are predicted to be secreted by the general secretion pathway (Sec), LipS1 contains an SP1 cleavage site, whereas *C. amycolatum* WP_005510233.1 contains an SPII cleavage site. Based on this analysis, we generated an in-frame deletion of WP_005510233.1 in *C. amycolatum*, referred to as Δ*lip C. amycolatum*, to determine whether this putative lipase contributes to *C. amycolatum*-mediated inhibition of *S. pneumoniae* growth. As *C. amycolatum* is non-lipophilic, Δ*lip C. amycolatum* grew similarly to WT in either Tween 80 or triolein (Supplementary Figure 7B). However, supernatant from Δ*lip C. amycolatum* no longer inhibited *S. pneumoniae* growth *in vitro* (Figure 6A), suggesting that this lipase is critical for *C. amycolatum*-mediated inhibition *in vitro*.

Lipases such as LipS1 and/or other bacterial factors may contribute to *Corynebacterium*-mediated protection against *S. pneumoniae in vivo*. Using our co-infection animal model, we compared the impact of pre-exposure to WT vs. Δ*lipS1 C. accolens* on *S. pneumoniae* colonization and infection. *S. pneumoniae* burdens in the upper airway (nasal lavage) were higher in mice pre-exposed to Δ*lipS1 C. accolens* compared to mice pre-exposed to WT *C. accolens* (Figure 6B). However, there was still a significant reduction in upper airway *S. pneumoniae* burdens in mice pre-exposed to Δ*lipS1 C. accolens* compared to mice infected with *S. pneumoniae* alone. These findings indicate that *C. accolens* expression of LipS1 contributes to, but is not solely required for, inhibition of *S. pneumoniae* colonization in the upper airway. In the lungs, mice pre-exposed to either Δ*lipS1* or WT *C. accolens* had significantly reduced burdens of *S. pneumoniae* compared with mice infected with *S. pneumoniae* alone (Figure 6B). There was a slight increase in the percentage of mice with detectable *S. pneumoniae* burdens in the lung following pre-exposure to Δ*lipS1 C. accolens* (38%) vs. WT *C. accolens* (15%), similar to the upper airway, however, this difference was not statistically significant. Burdens of WT and Δ*lipS1 C. accolens* in the nasal lavage and lungs of co-infected mice were similar (Figure 6C), indicating no colonization defect for Δ*lipS1 C. accolens*. Also, mice with the highest burdens of either WT or Δ*lipS1 C. accolens* correlated with lower and frequently undetectable burdens of *S. pneumoniae* in the lung (Supplementary Figure 5C), confirming a relationship between *C. accolens* abundance and reduced *S. pneumoniae* infection of the lung. These data indicate that multiple mechanisms, both LipS1-dependent and independent, contribute to *Corynebacterium*-mediated protection against *S. pneumoniae in vivo*.

## Discussion

Our findings provide novel evidence in support of the potential benefit of *Corynebacterium* abundance in the airway. Both *C. accolens* and *C. amycolatum* inhibited *S. pneumoniae* growth *in vitro*, observations corroborated by protection *in vivo* using a new co-infection model. The only mechanism for *Corynebacterium*-mediated protection against *S. pneumoniae* described thus far is the liberation of host FAs by LipS1 in *C. accolens* (Bomar et al., 2016), as confirmed herein both *in vitro* and *in vivo*. Beyond direct killing of *S. pneumoniae*, FAs induce expression of the antimicrobial peptide β-defensin-2 by human sebocytes (Nakatsuji et al., 2010), which may further enhance protection against *S. pneumoniae* in the airway. We demonstrate that FAs are present in the upper airway of mice, as in humans (Do et al., 2008), consistent with their availability to serve as a nutrient source for lipophilic *Corynebacterium* such as *C. accolens*. The finding that *Corynebacterium* colonization did not influence FA abundance may result from a lack of accessible triacylglycerols for FA production, as has been suggested (Bomar et al., 2016), metabolic turnover, or temporal fluctuations which were missed. Alternatively, we did not determine whether the expression of LipS1 is upregulated in co-infected mice. Regardless, the observation that LipS1-deficient *C. accolens* is less protective against *S. pneumoniae* suggests that lipase activity contributes to *S. pneumoniae* killing *in vivo*. Others have confirmed that the mouse airway can be colonized with endogenous *Corynebacterium*, which becomes the dominant genus following treatment with the antibiotic neomycin (Ichinohe et al., 2011). This finding indicates that competition with the resident microbiota limits *Corynebacterium* colonization, consistent with our inability to establish *Corynebacterium* colonization in non-antibiotic-treated mice (not shown). Thus, while host lipids, including FAs, are present in the mouse airway, microbial depletion is likely a requirement for modeling *Corynebacterium* colonization.

The partial protective effect of LipS1-deficient *C. accolens* indicates a role for additional mechanisms of *Corynebacterium*-mediated protection *in vivo*. *S. pneumoniae* and other opportunistic pathogens are enriched following antibiotic therapy in children, whereas *Corynebacterium* are reduced (Teo et al., 2015; Kelly et al., 2021), suggesting that *S. pneumoniae* is better equipped for expansion into the microbiome-depleted airway. We found that colonizing mice with *Corynebacterium* prior to *S. pneumoniae* exposure is sufficient to reverse this expansion. In addition to competition with pathogens through the expression of bacterial lipases, some *Corynebacterium* species produce siderophores, which reduce growth of the pathogen *Staphylococcus aureus* in iron-limiting environments (Stubbendieck et al., 2019), although the potential impact on *S. pneumoniae* is not known. Beyond competition for niche space and nutrients, interactions between *Corynebacterium* and airway epithelial cells may contribute to enhanced protection against *S. pneumoniae in vivo*. For example, some species of *Corynebacterium* express the molecule phosphorylcholine (ChoP) (Gillespie et al., 1996), which binds to platelet activating factor receptor (PAFR), a critical adherence receptor for *S. pneumoniae* (Rijneveld et al., 2004). ChoP expression by selected *Corynebacterium* species may therefore interfere with *S. pneumoniae* access to PAFR, reducing *S. pneumoniae* acquisition and infection. Moreover, *Corynebacterium* activation of airway epithelial cells may enhance their production of antimicrobial peptides in a FA-independent manner, as with other airway commensals (Liu et al., 2019). Finally, *Corynebacterium*-mediated recruitment of inflammatory myeloid cells, as we observed in the lung, may serve as an immune priming signal to enhance clearance of *S. pneumoniae*, as neutrophils and inflammatory monocytes are critical for early *S. pneumoniae* killing (Calbo and Garau, 2010). The development of an animal model for *Corynebacterium* colonization, which mirrors the relationship between *Corynebacterium* abundance and risk of *S. pneumoniae* acquisition observed in humans, should facilitate investigation of these and other mechanisms in future studies. However, translation of such findings in mice into novel therapeutic approaches will require verification that similar effects are active during human colonization with *Corynebacterium*.

In these studies, two morphologically and metabolically distinct species of *Corynebacterium* protected against *S. pneumoniae* growth. The non-lipophilic *C. amycolatum* was only inhibitory *in vitro* upon the addition of multiple FA sources, indicating that screens which do not incorporate enough exogenous lipids may miss the inhibitory potential of additional *Corynebacterium* clinical isolates. Indeed, while airway microbiome sequencing studies indicate a broadly protective role for *Corynebacterium* species against respiratory tract infections and *S. pneumoniae*, mechanistic studies have revealed heterogeneity among different *Corynebacterium*-pathogen pairs. For example, both *C. pseudodiphtheriticum* and *Corynebacterium striatum* inhibit growth of the pathogen *S. aureus*, whereas *C. accolens* instead promotes *S. aureus* growth *in vitro* (Yan et al., 2013; Ramsey et al., 2016). *C. pseudodiphtheriticum* mediates a broadly protective effect against primary and secondary *S. pneumoniae* infections (Kanmani et al., 2017; Moyano et al., 2020) and also inhibits growth of another pathogen, *Moraxella catarrhalis*, *in vitro* (Lappan and Peacock, 2019). The potential therapeutic effect of *C. pseudodiphtheriticum* has been evaluated in humans, where delivery by nasal spray was shown to reduce colonization with *S. aureus* in healthy adults (Kiryukhina et al., 2013). While this was only tested in four subjects, another *Corynebacterium* designated as strain Co304 had a similar effect in another 17 subjects (Uehara et al., 2000). The impact of other *Corynebacterium* species, including those such as *C. accolens* which may inhibit some pathogens but promote others, on a broader infection profile will be important to determine in future studies.

Colonization of mice by both species of *Corynebacterium* (*C. accolens* and *C. amycolatum*) elicited comparably robust myeloid cell responses in the lung in parallel with elevations in the chemotactic protein MCP-1. However, neither species induced appreciable expression of pro-inflammatory cytokines in lung BAL. As reported following colonization with *C. pseudodiphtheriticum* in infant mice (Kanmani et al., 2017), both TNFα and IFNγ were increased in the serum, as in our study, but, unlike our results, several pro-inflammatory cytokines were also increased in the BAL, highlighting the importance of investigating species-specific outcomes. Colonization at different host sites may also induce distinct immune activation profiles. We did not detect an IL-17A response in the lungs of *Corynebacterium*-exposed mice, whereas skin colonization with *C. accolens*, but not *C. amycolatum*, was associated with the induction of a γδT cell IL-17A response (Ridaura et al., 2018). The lung is particularly sensitive to the detrimental effects of inflammation, which causes damage to the integrity of the barrier, reducing oxygen exchange (Muller-Redetzky et al., 2014). *S. pneumoniae* co-infections, particularly with viral pathogens, are associated with an enhanced inflammatory response, promoting inflammation-mediated damage in the co-infected lung and leading to increased morbidity and mortality (Kash et al., 2011; Aguilera and Lenz, 2020). In contrast, we find that, unlike with respiratory viral and other pathogens, co-infection with *Corynebacterium* tempers the inflammatory response to *S. pneumoniae*, as described for *C. pseudodiphtheriticum* in the context of sterile lung injury (Kanmani et al., 2017; Moyano et al., 2020), along with *S. pneumoniae* clearance, indicating the potential to improve lung recovery from infection and injury.

A limitation of this study is that we did not investigate co-colonization with *Dolosigranulum*, which frequently associates with *Corynebacterium* in the human upper airway of humans (Bogaert et al., 2011; Laufer et al., 2011; Copeland et al., 2018). Abundances of several different *Corynebacterium* species are increased in both child and adult carriers of *Dolosigranulum* (Brugger et al., 2020). *In vitro*, these authors demonstrated that some *Corynebacterium* species support growth of *Dolosigranulum pigrum*, although not consistently (Brugger et al., 2020). Further, *S. pneumoniae* growth was inhibited *in vitro* by *D. pigrum* and *C. pseudodiphtheriticum*, neither of which were inhibitory on their own under the assay conditions used (Brugger et al., 2020). This study alludes to greater complexity in the human airway, where *Corynebacterium*-pathogen interactions are likely influenced by the presence of other commensal species including *Dolosigranulum*. In addition, *Corynebacterium* and *Dolosigranulum* must themselves compete with other airway commensals. For example, *Staphylococcus epidermidis* can inhibit both *D. pilgrim* and *C. pseudodiphtheriticum* growth *in vitro* (Janek et al., 2016). These findings highlight the importance of considering multiple commensal-pathogen dynamics in the airway, beyond interactions between specific commensal-pathogen pairs.

In summary, these studies identify *C. accolens* and *C. amycolatum* as *Corynebacterium* species which are capable of short-term colonization in antibiotic treated mice. Further, pre-exposure to either of these *Corynebacterium* species significantly reduces *S. pneumoniae* infection while mitigating lung inflammation. Although the host and bacterial requirements for *Corynebacterium*-mediated protection against *S. pneumoniae* are not fully resolved, both *C. accolens* and *C. amycolatum* express bacterial lipases which contribute to growth inhibition *in vitro*, a mechanism responsible, in part, for *C. accolens*-mediated protection *in vivo*. These findings indicate that therapeutic strategies to enhance *Corynebacterium* colonization may reduce the risk of *S. pneumoniae* acquisition and infection. We envision that such approaches would be particularly beneficial following antibiotic therapy, when the endogenous airway microbiome is disrupted. However, it is also important to consider potential adverse effects associated with enhanced *Corynebacterium* colonization, as some species may serve as a source of opportunistic infections. Improved understanding of the mechanisms of *Corynebacterium*-mediated protection will therefore facilitate harnessing the potential therapeutic benefit of these endogenous “protector” bacteria.

## Data Availability Statement

The original contributions presented in the study are included in the article/Supplementary Material, further inquiries can be directed to the corresponding author/s.

## Ethics Statement

Animal studies were reviewed and approved by the Animal Care and Use Committee of the University of Colorado School of Medicine.

## Author Contributions

SC conceived the study and designed the experiments together with VA, EJ, and SA. SC, KH, VA, and SA performed the experiments. SC wrote the manuscript. SC and EJ edited the manuscript. All authors contributed to the article and approved the submitted version.

## Conflict of Interest

The authors declare that the research was conducted in the absence of any commercial or financial relationships that could be construed as a potential conflict of interest.

## Publisher’s Note

All claims expressed in this article are solely those of the authors and do not necessarily represent those of their affiliated organizations, or those of the publisher, the editors and the reviewers. Any product that may be evaluated in this article, or claim that may be made by its manufacturer, is not guaranteed or endorsed by the publisher.

## Footnotes

1
https://www.addgene.org/110086/
