# Supplementary material for: Corynebacterium Species Inhibit Streptococcus pneumoniae Colonization and Infection of the Mouse Airway
Source: Front Microbiol. 2022 Jan 10;12:804935. doi: 10.3389/fmicb.2021.804935 (PMC8784410; doi:10.3389/fmicb.2021.804935)
Supplement: Supplementary file 1 [file Data_Sheet_1.pdf]

Supplementary Material

Supplementary Table 1. Primers used in this study.

Primer	5' to 3' Sequence	Use	Reference
KL516	gcggatccagctgcagCCGCCGGCAATTCGCCTG	Gibson assembly for *lipS1* deletion in *C. acc*	Bomar et al., 2016
KL517	gggtgccctcgagtgCATGAGAACCCCTGAAGCCACAAG	Gibson assembly for *lipS1* deletion in *C. acc*	Bomar et al., 2016
KL518	gttctcatgcactcgagggcacccggagctTAAATACTGCTCCCTTACGCC	Gibson assembly for *lipS1* deletion in *C. acc*	Bomar et al., 2016
KL519	aagcttcgaattctgcaGGTTGGGTTCGATGCGCT	Gibson assembly for *lipS1* deletion in *C. acc*	Bomar et al., 2016
KL516b	gcggatccagctgcagCCGACGACTTCGACGTACAC	Gibson assembly for *lip* deletion in *C. amy*	This study
KL517b	gggtgccctcgagtgCCATGCTCAGCGCAATTCTG	Gibson assembly for *lip* deletion in *C. amy*	This study
KL518b	gttctcatgcactcgagggcacccggagctCGCACTAGCGCCCTAGCTGTA	Gibson assembly for *lip* deletion in *C. amy*	This study
KL519b	aagcttcgaattctgcaGATCCGCGTCGTGGTGAAC	Gibson assembly for *lip* deletion in *C. amy*	This study
pKOIF	TGCCTCTATGCGCGATGATT	Amplifying fragment insertion site from pKO	This study
pKOIR	TTCACGACGAGAGGAGACCT	Amplifying fragment insertion site from pKO	This study
KL556	ACTGGAGCTTCCTCATCGTT	Sequencing *lipS1* mutant in *C. acc*	Bomar et al., 2016
KL557	TTGAAAACAGGATGCGACGAT	Sequencing *lipS1* mutant in *C. acc*	Bomar et al., 2016
KL556b	ATTCGAACAAGTGCGTTGCC	Sequencing *lip* mutant in *C. amy*	This study
KL557b	AAGCGTATCGGTCCAGTGAC	Sequencing *lip* mutant in *C. amy*	This study
16SF	ACTCCTACGGGAGGCAGCAGT	16S rRNA qPCR	Jimeno et al., 2018
16SR	ATTACCGCGGCTGCTGGC	16S rRNA qPCR	Jimeno et al., 2018

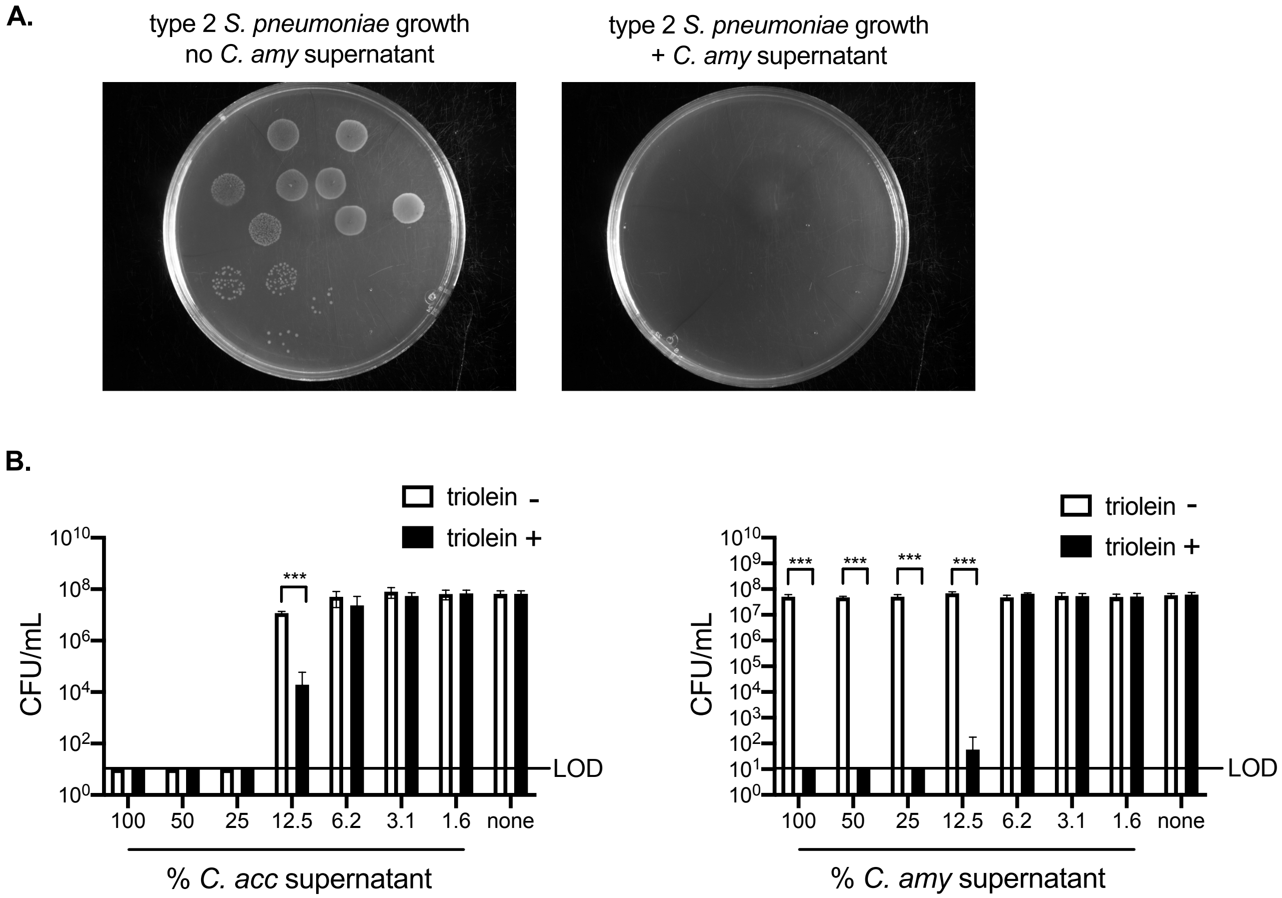

Supplementary Figure 1. Inhibition of *S. pneumoniae* growth *in vitro* by *Corynebacterium* species (related to Figure 1). Representative photographs of serial dilution spot plating of type 2 *S. pneumoniae* (10 µL per dilution) on untreated plates versus plates pre-treated with supernatant from *C. amycolatum* (*C. amy*) (**A**). Growth of type 2 *S. pneumoniae* following the addition of 100-1.6% supernatants from *C. accolens* (*C. acc*) or *C. amy* from BHI supplemented with 1% Tween 80 with or without 180 mg/mL triolein (**B**). None indicates the addition of BHI broth supplemented with 1% Tween 80, with or without triolein. Limit of detection (LOD) is indicated. Data are pooled from three experiments in duplicate. ***p<.001, ANOVA.

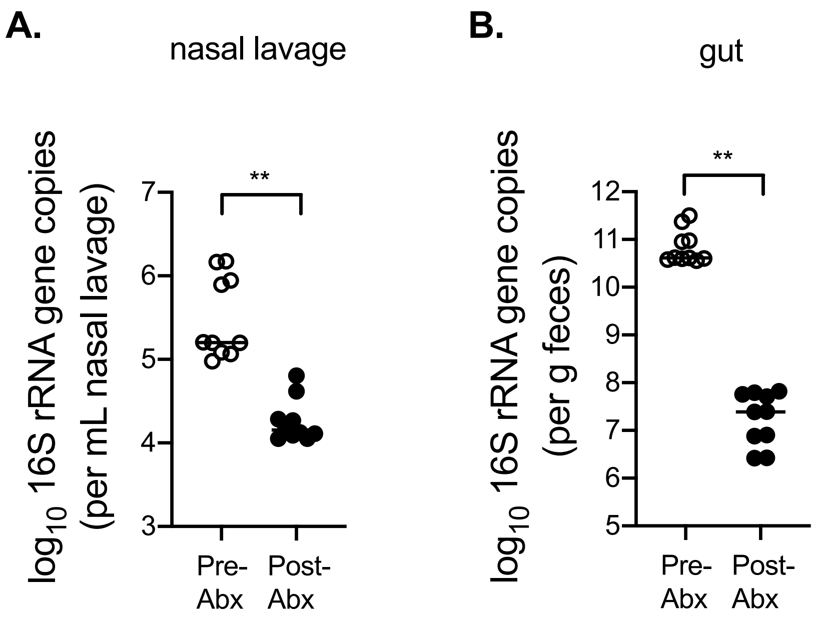

Supplementary Figure 2. Microbiome depletion in antibiotic treated mice (related to Figure 2). 16S rRNA gene copies per mL in the nasal lavage fluid of naïve mice (pre-Abx) or mice following 7 days on water containing an antibiotic cocktail (post-Abx) detected by qPCR (**A**). 16S rRNA gene copies per gram of feces from naïve mice (pre-Abx) or the same mice following 7 days on water containing an antibiotic cocktail (post-Abx) detected by qPCR (**B**). Data are pooled from three independent experiments with 3-4 mice per group. **p<.01, ***p<.001, t test.

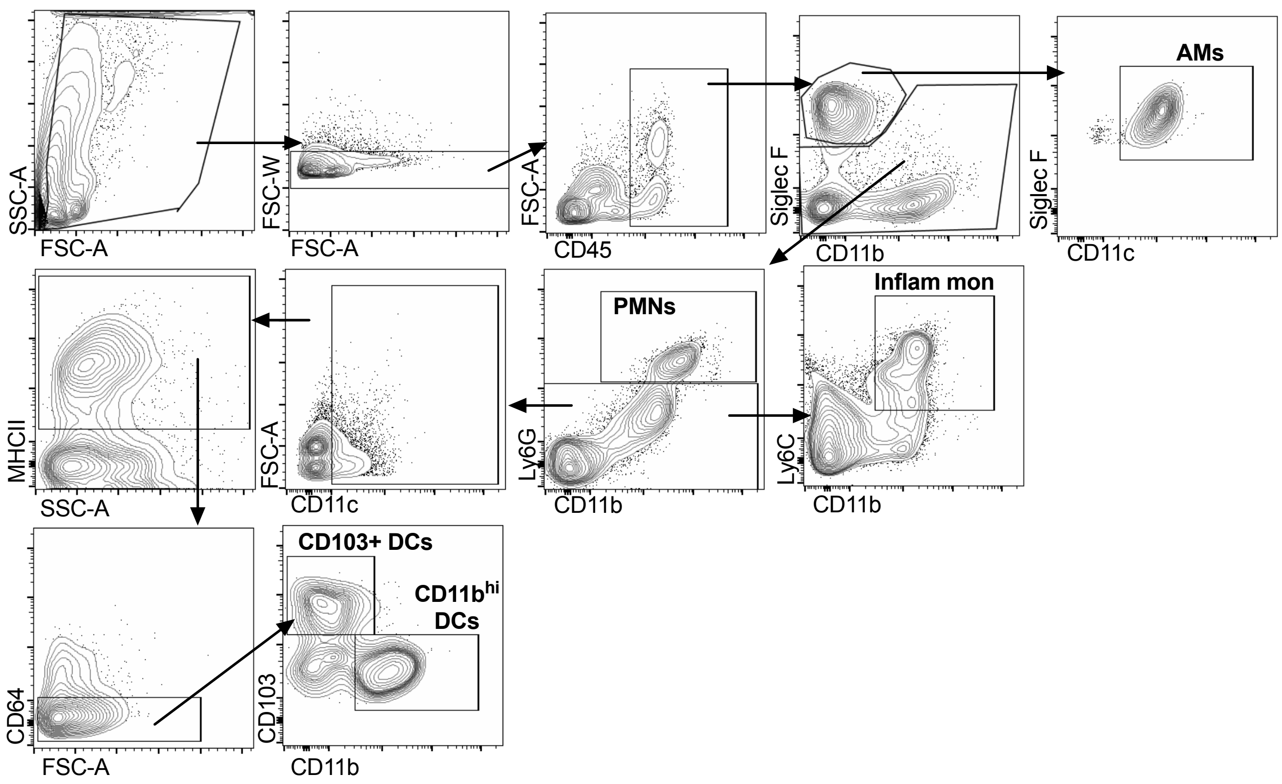

Supplementary Figure 3. Detection of innate immune cell populations in the lung by flow cytometry (related to Figure 3). Representative flow plots demonstrating the gating strategy used to identify neutrophils (PMNs), inflammatory monocytes (Inflam mon), alveolar macrophages (AMs), CD103^{+} dendritic cells (DCs), and CD11b^{hi} DCs from single cell preparations from the lungs of mice.

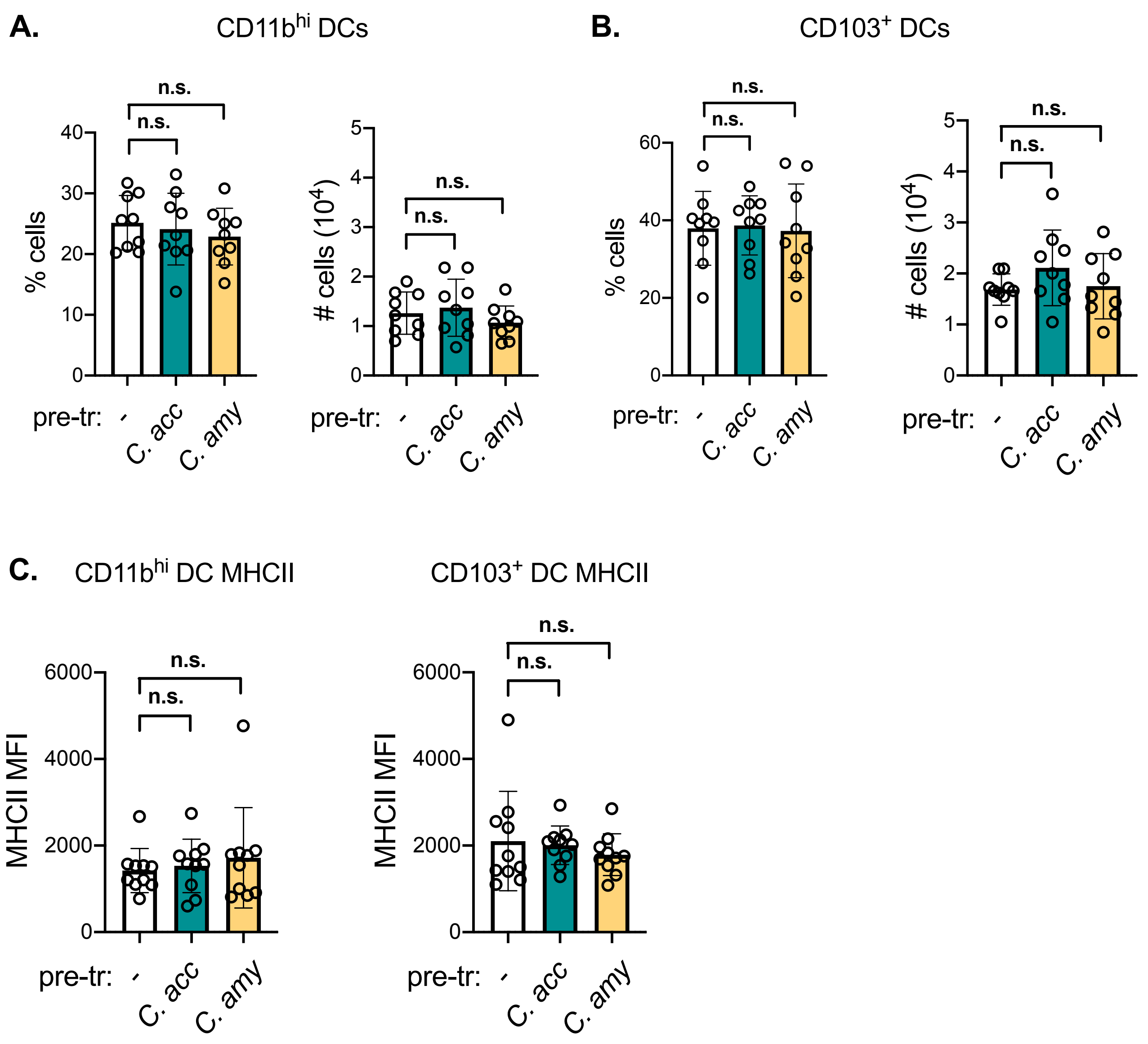

Supplementary Figure 4. Dendritic cells in the lungs of *Corynebacterium* colonized mice (related to Figure 3). Percentage and total number of CD11b^{hi} dendritic cells (DCs) (**A**) and CD103^{+} DCs (**B**) and as well as MHCII mean fluorescence intensity (MFI) in these cell populations (**C**) detected by flow cytometry in the lungs of naïve mice and mice 48 hours post-infection with *C. accolens* (*C. acc*), *C. amycolatum* (*C. amy*), or PBS (-). Data are pooled from three independent experiments with 3-6 mice per group. No significant differences detected between groups, ANOVA.

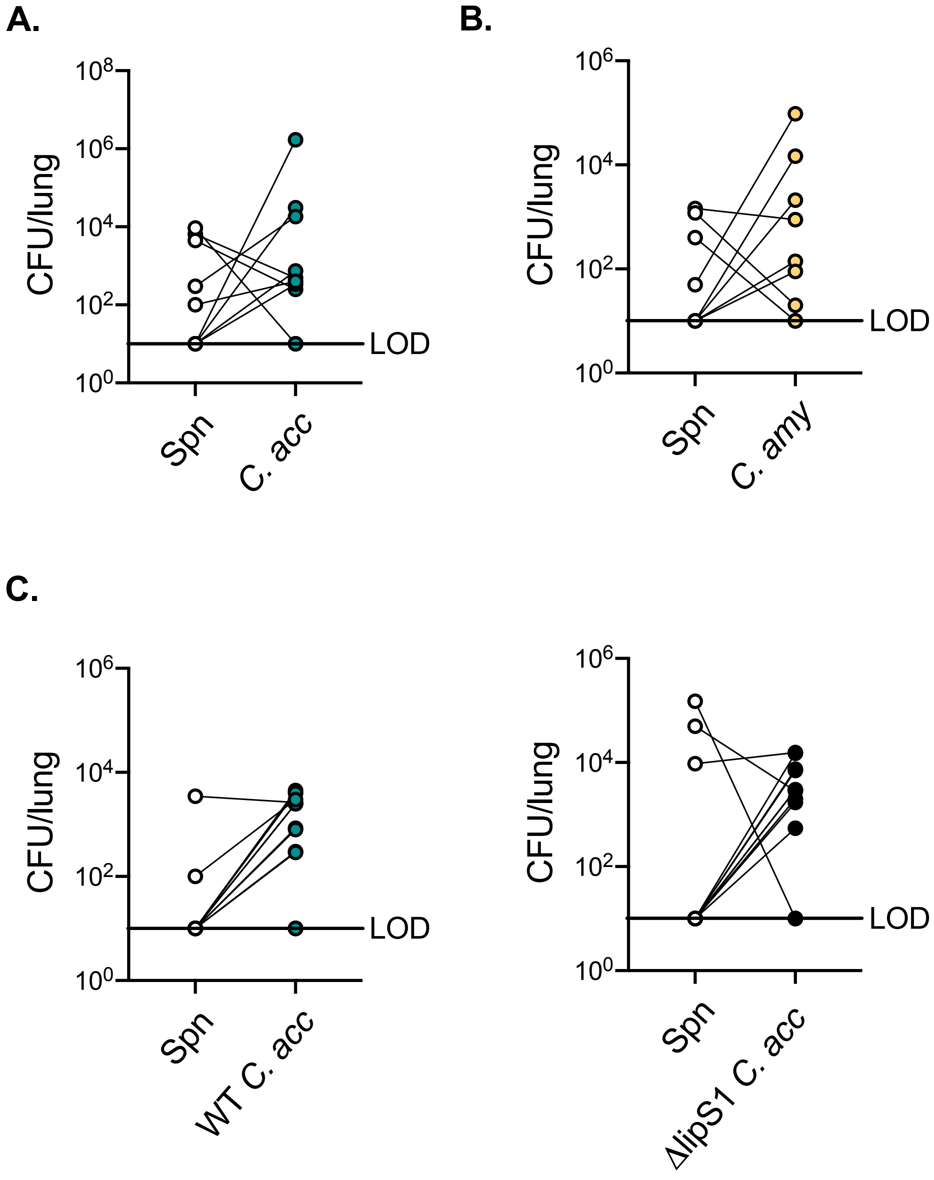

Supplementary Figure 5. Relationship between *S. pneumoniae* and *Corynebacterium* burdens in co-infected mice (related to Figures 4 and 6). Burdens of *S. pneumoniae* (Spn) versus burdens of *C. acc* (**A**) or *C. amy* (**B**) in the lungs of co-infected mice at 24 hours post-Spn infection detected by live growth (from data shown in Figure 4), with lines indicating burdens derived from the same individual mice. Burdens of Spn versus burdens of WT or ΔlipS1 *C. acc* (**C**) in the lungs of co-infected mice at 24 hours post-Spn infection detected by live growth (from data shown in Figure 6), with lines indicating burdens derived from the same individual mice. Limit of detection (LOD) is indicated. Data are pooled from three independent experiments with 3-5 mice per group.

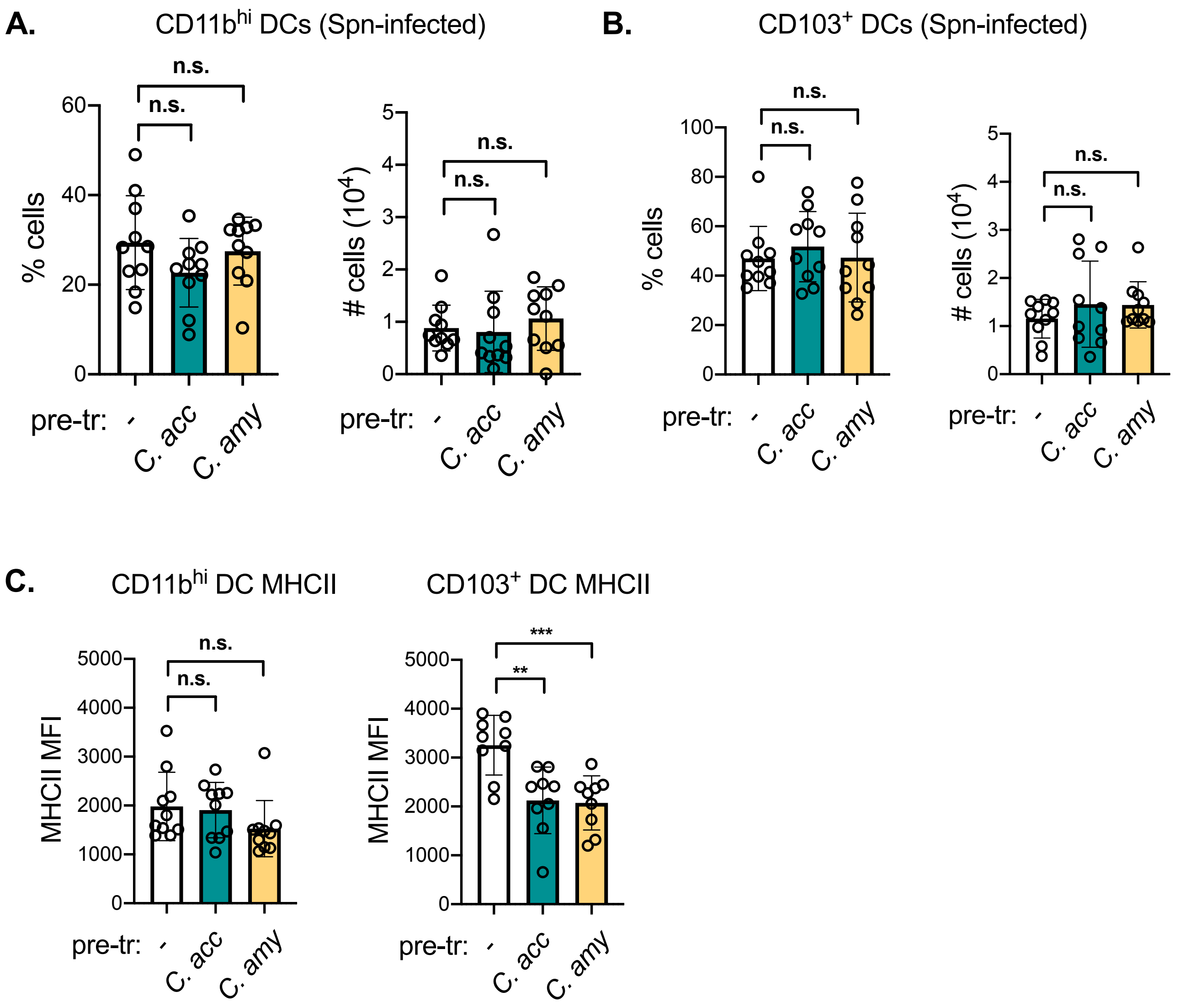

Supplementary Figure 6. Dendritic cells in the lungs of *Corynebacterium-S. pneumoniae* co-infected mice (related to Figure 5). Percentage and total number of CD11b^{hi} DCs (**A**) and CD103^{+} DCs (**B**) as well as MHCII mean fluorescence intensity (MFI) in these cell populations (**C**) detected by flow cytometry in the lungs of mice pre-exposed to PBS (-), *C. acc* or *C. amy* at 24 hours post-*S. pneumoniae* (Spn) infection. Data are pooled from three independent experiments with 3-6 mice per group. **p<.01, ***p<.001, ANOVA.

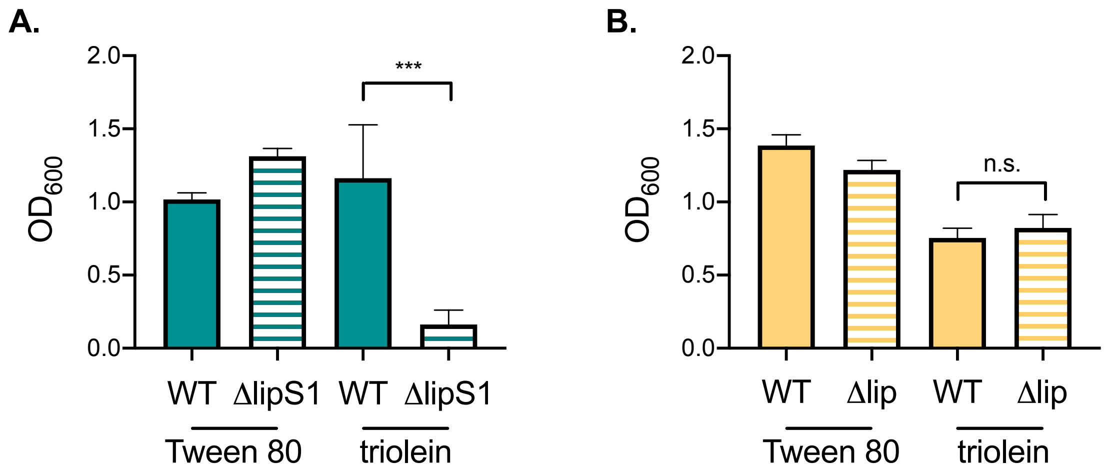

Supplementary Figure 7. Growth of Δ*lipS1 C. accolens*, but not Δ*lip C. amycolatum*, is deficient with triolein as the sole source of FAs (related to Figure 6). Turbidity (OD_{600}) in BHI broth cultures supplemented with or without 1% Tween 80 and 180 mg/mL triolein following 8 hours of aerobic growth for WT versus Δ*lipS1 C. accolens* (*C. acc*) (**A**) and WT versus Δ*lip C. amycolatum* (*C. amy*) (**B**). Data are pooled from three independent experiments, run in duplicate. ***p<.001, t test.
